# Laboratory divergence of *Methylobacterium extorquens* AM1 through unintended domestication and past selection for antibiotic resistance

**DOI:** 10.1186/1471-2180-14-2

**Published:** 2014-01-02

**Authors:** Sean Michael Carroll, Katherine S Xue, Christopher J Marx

**Affiliations:** 1 Department of Organismic and Evolutionary Biology, Harvard University, Cambridge, MA, USA; 2 Chemical and Physical Biology, Harvard College, Cambridge, MA, USA; 3 Faculty of Arts and Sciences Center for Systems Biology, Harvard University, Cambridge, MA, USA

**Keywords:** Laboratory domestication, *Methylobacterium extorquens* AM1, Antibiotic resistance, Rifamycin, Whole-genome sequencing, Strain integrity

## Abstract

**Background:**

A common assumption of microorganisms is that laboratory stocks will remain genetically and phenotypically constant over time, and across laboratories. It is becoming increasingly clear, however, that mutations can ruin strain integrity and drive the divergence or “domestication” of stocks. Since its discovery in 1960, a stock of *Methylobacterium extorquens* AM1 (“AM1”) has remained in the lab, propagated across numerous growth and storage conditions, researchers, and facilities. To explore the extent to which this lineage has diverged, we compared our own “Modern” stock of AM1 to a sample archived at a culture stock center shortly after the strain’s discovery. Stored as a lyophilized sample, we hypothesized that this Archival strain would better reflect the first-ever isolate of AM1 and reveal ways in which our Modern stock has changed through laboratory domestication or other means.

**Results:**

Using whole-genome re-sequencing, we identified some 29 mutations – including single nucleotide polymorphisms, small indels, the insertion of mobile elements, and the loss of roughly 36 kb of DNA - that arose in the laboratory-maintained Modern lineage. Contrary to our expectations, Modern was both slower and less fit than Archival across a variety of growth substrates, and showed no improvement during long-term growth and storage. Modern did, however, outperform Archival during growth on nutrient broth, and in resistance to rifamycin, which was selected for by researchers in the 1980s. Recapitulating selection for rifamycin resistance in replicate Archival populations showed that mutations to RNA polymerase B (*rpoB*) substantially decrease growth in the absence of antibiotic, offering an explanation for slower growth in Modern stocks. Given the large number of genomic changes arising from domestication (28), it is somewhat surprising that the single other mutation attributed to purposeful laboratory selection accounts for much of the phenotypic divergence between strains.

**Conclusions:**

These results highlight the surprising degree to which AM1 has diverged through a combination of unintended laboratory domestication and purposeful selection for rifamycin resistance. Instances of strain divergence are important, not only to ensure consistency of experimental results, but also to explore how microbes in the lab diverge from one another and from their wild counterparts.

## Background

To ensure that scientific results are both reproducible and consistent, a high level of integrity is required of experimental methods and microbial stocks. One assumption is that stocks are constant over time, such that contemporary isolates of a given strain are genetically and physiologically identical across laboratories, and to stocks from many years ago. However, before the widespread use of deep freezers or lyophilization, storage of stocks using agar slants and other methods permitted growth and metabolism, albeit slowly, over long periods of time. And as long as stocks are metabolically active, mutations are likely to appear. If these mutations are beneficial, they can be enriched or fixed in stocks and subsequent sub-cultures via natural selection; or, alternatively, practices such as plate streaking and colony picking could inadvertently propagate clones with neutral or even deleterious mutations due to random sampling or “genetic drift”. Either way, such mutations are the bane of microbial stocks: they destroy the integrity of otherwise isogenic lines, and they become the raw material for processes such as selection and drift to facilitate evolutionary divergence in strains over time.

The slow accrual of mutations often goes unnoticed in laboratory strains, but can result in considerable genomic and phenotypic differences both between independent laboratory stocks, and between laboratory strains and their wild counterparts. This unintended mutational divergence is termed “laboratory domestication”, and is particularly common in many (if not most) microorganisms isolated prior to the widespread use of modern storage methods (e.g., cryopreservation in deep freezers or lyophilization). For example, the domestication of *Bacillus subtilis* to the lab is associated with a loss of biofilms [1], swarming behavior [2], and fruiting body formation (sporulation) [3]. Stocks of *Salmonella enterica* serovar Typhimurium [4,5] and *Escherichia coli*[6-8] archived for years or decades show a considerable amount of genetic diversity and novel phenotypes stemming from long-term growth and survival during storage in agar stabs. Other controlled studies of microbes from the lab [9,10] or the wild [11-13] also show how growth, storage, and passaging procedures can readily lead to the diversification, divergence, and domestication of stocks. Outside of microbes, examples of domestication have been documented in stocks of the nematode worm, *C. elegans*[14,15], and in independent stocks of laboratory mice [16,17]. In all these examples, understanding the extent to which laboratory domestication has occurred is important - not only for the standardization of experiments and the correct interpretation of results - but also because each instance of domestication is itself an interesting case-study of genomic and phenotypic divergence driven by a subtle and often cryptic set of evolutionary processes.

Since the early 1960s, *Methylobacterium extorquens* AM1 has emerged as the predominant model system for studies of bacterial one-carbon metabolism. As a facultative methylotroph, *M. extorquens* AM1 (herein referred to as “AM1”) has the ability to grow using reduced one-carbon (C_1_) compounds such as methanol and methylamine as the sole source of carbon and energy, as well as multi-carbon (multi-C) compounds such as succinate, pyruvate, and acetate [18,19]. The oxidation of methanol into biomass proceeds via the highly toxic intermediate, formaldehyde, and is complex, requiring over 100 enzymes [20]. A sequenced genome [21], genetic tools [22-26], optimized growth conditions [27], metabolic models [28], and extensive knowledge of both C_1_ and multi-C metabolism [29] all make AM1 the ideal organism for studies of methylotrophy in the lab, as well as an emerging system for experimental evolution [30-33]. Aside from AM1, related methylobacteria are known for their roles in the plant microbiome [34-37], the biodegradation of toxic chemicals like chloromethane [38] and dichloromethane [39], and their potential for use in industrial applications [40,41].

Unlike most organisms, the discovery and establishment of AM1 as a model system was completely accidental. Around the year 1960, Dr. J.R. Quayle and colleagues at the University of Oxford were searching for a new organism in which to study the oxidation and assimilation of C_1_ compounds, but discovered in their medium a “heavy, pink growth, presumably due to some airborne contaminant” [18,42]. After the contaminant was isolated, it was found to grow rapidly on a variety of both C_1_ and multi-C compounds. Shortly thereafter, a sample of AM1 - then known as *Pseudomonas* AM1 for “**A**irborne **M**ethylotroph #**1**” - was deposited to the National Collection of Industrial and Marine Bacteria (NCIMB, Aberdeen, Scotland), while a working stock remained in the lab. Over the years, this working stock was maintained and propagated between different researchers, laboratories, and growth and storage conditions, all the way up to our own laboratory’s stock. These circumstances raise the question: to what extent has this AM1 lineage diverged during its time in the lab?

**Figure 1:**
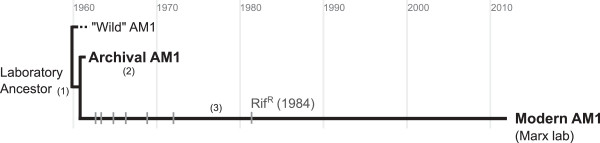
**Two distinct lineages of *Methylobacterium extorquens *AM1.** Shortly after its discovery in 1960 (1), a sample of *M. extorquens* AM1 (“AM1”) was deposited to a culture stock center for storage and distribution (2; Archival AM1). Many researchers, however, use instead a working stock of AM1 that was maintained over fifty years in the lab (3; Modern AM1), and was at one point selected for rifamycin resistance (Rif^R^) [45]. We hypothesized that these conditions may have fostered the accumulation of mutations and unintended evolutionary divergence in the Modern AM1 lineage, and sought to compare our Modern AM1 to the Archival strain. Dashes represent the accumulation of mutations in the Modern lineage.

To address this question, we sought to compare today’s AM1 to its ancestor isolated circa 1960, or a close descendent of this ancestor. Closely related strains of *M. extorquens* differ significantly in their gene content and metabolic capabilities [43], making these a less than ideal comparison to determine the ancestral “wild” AM1 state. Luckily, however, we realized that two major lineages of AM1 were established circa 1960: the stock archived circa 1961 at the culture stock center (herein referred to as the “Archival” strain); and the working line of AM1 that was propagated over many years from J.R. Quayle’s lab, to Mary Lidstrom’s group, to our own laboratory stock of “Modern” AM1 (Figure 1). We hypothesized that the Archival AM1 – which underwent longer periods of lyophilized storage with fewer growth cycles in between – might better-reflect the ancestral state of AM1 circa 1960, and offer an excellent reference with which to identify evolutionary divergence in the laboratory-maintained AM1 lineage. We document here the surprising extent to which our Modern AM1 has changed during its time in the lab using various assays of growth and fitness, long-term growth and storage, and whole-genome sequencing of the Archival AM1 strain. We then offer a discussion of specific laboratory practices and evolutionary processes that may have led to such divergence.

## Results

### Whole-genome sequencing reveals extensive genomic divergence in the Modern AM1 lineage

To explore the extent to which AM1 has diverged at the genomic level, we used whole-genome sequencing to compare the Archival genome to a previously sequenced Modern reference [21]. Illumina sequencing reads were analyzed both by mapping onto the Modern reference, and through *de novo* sequence assembly. For sites in which these strains differed, we compared the mutational state at each site (i.e., Archival or Modern) to other previously sequenced strains of *M. extorquens* to determine whether substitutions occurred in either the Modern or Archival lineage (Figure 1). While this analysis cannot identify changes that occurred between the divergence of “wild” AM1 from the Archival/Modern laboratory ancestor, these mutations, if any, would only add to the ways in which AM1 has evolved in the lab.

Our results identified a sizeable number and variety of mutations that separate the Modern and Archival strains. We discovered 11 SNPs, 4 small indels (1–5 bp), the proliferation of 9 insertion sequence (mobile) elements, and some 36 kb of DNA found in 5 *de novo* assembled contigs that are present in the Archival strain but absent in Modern AM1 (Table 1). For all but two of these mutations, excluding DNA loss, the Archival state is universally conserved with related *M. extorquens* strains to the exclusion of Modern AM1. Taken together, these results strongly suggest that this extensive genomic divergence occurred solely in the Modern AM1 lineage, while the Archival AM1 has been largely preserved.

**Table 1:** List of mutations derived in the Modern AM1 lineage

**Single nucleotide polymorphisms**					
**Chromosome**	**Position**	**Mutation**	**Annotation**	**Gene/Locus**	**Description**
META1	611,700	G → A	Intergenic (+342/-18)	*META1_0578/META1_0579*	Transcriptional accessory protein/hypothetical protein
META1	2,050,899	G → C	S359R (AG ***G*** → AG ***C***)	*META1_1984*	Putative catecholate siderophore receptor fiu precursor (TonB-dependent receptor fiu)
META1	2,511,236	C → T	L8004F (***C*** TC → ***T*** TC)	*META1_2412*	Hypothetical protein
META1	3,795,848	C → T	L264F (***C*** TC → ***T*** TC)	*aldB*	Aldehyde dehydrogenase; chloroacetaldehyde dehydrogenase
META1	4,123,848	C → T	Intergenic (-1754/+2180)	*META1_4038/rffH*	Fragment of transposase related to IS701 family/glucose-1-phosphate thymidylyltransferase
META1	4,382,526	C → G	T160T (AC ***C*** → AC ***G***)	*META1_4292*	Plasmid replication protein RepA
META1	4,494,119	A → G	N55D (***A*** AC → ***G*** AC)	*rplJ*	50S ribosomal subunit protein L10
META1	4,496,733	A → G	Q521R (C ***A*** G → C ***G*** G)	*rpoB*	RNA polymerase, beta subunit
META1	4,498,413	A → G	Q1081R (C ***A*** G → C ***G*** G)	*rpoB*	RNA polymerase, beta subunit
META1	4,665,003	C → A	L300M (***C*** TG → ***A*** TG)	*recG*	DNA helicase, ATP-dependent resolution of Holliday junctions, branch migration
META1	5,187,175	G → A	A260T (***G*** CC → ***A*** CC)	*META1_5044*	Putative o-succinylbenzoate--CoA ligase
**Small insertions & deletions**					
**Chromosome**	**Position**	**Mutation**	**Annotation**	**Gene/Locus**	**Description**
META1	1,083,921	+1 bp	Intergenic (-299/+188)	*META1_1041/META1_1042*	Putative CoxB/conserved hypothetical protein
META1	2,012,135	+5 bp	Coding (126-130/228 nt)	*META1_1939*	Hypothetical protein
META1	5,018,430	Δ1 bp	Intergenic (-120/+218)	*META1_4900/META1_4901*	Putative hydrolase of beta-lactamase superfamily/conserved hypothetical protein DUF949
META2	821,910	+1 bp	Intergenic (+133/-26)	*META2_0863/META2_0864*	AAA superfamily ATPase/hypothetical protein
**IS elements**					
**Chromosome**	**Position**	**Mutation**	**Annotation**	**Gene/Locus**	**Description**
META1	772,350	+1408 bp	Gain ISMex3; coding	*META1_0742–META1_0743*	*META1_0742, META1_0743*
META1	929,023	+1390 bp	Gain ISMex14; intergenic	*META1_0895-META1_0898*	Transposase of ISMex1, IS3 family (ORF 1)/transposase of ISMex14, IS256 family
META1	3,730,805	+1408 bp	Gain ISMex3; coding	*META1_3592–META1_CDS3732187D*	*META1_3592, META1_3593, META1_CDS3732187D*
META1	4,143,329	+1620 bp	Gain ISMex4; intergenic	*META1_4059-META1_4061*	Hypothetical protein/transposase of ISMex4, IS1380 family
META1	4,149,803	+1408 bp	Gain ISMex3; coding	*META1_4069*	*META1_4069*
META1	4,702,223	+1620 bp	Gain ISMex4; intergenic	*META1_4586-META1_CDS4704205D*	Transposase of ISMex4, IS1380 family/hypothetical protein; RMQ08497
META1	4,909,262	+1620 bp	Gain ISMex4; coding	*META1_4801-META1_4803*	Hypothetical protein///transposase of ISMex4, IS1380 family/conserved hypothetical protein
META2	426,304	+1205 bp	Gain ISMex1; coding	*META2_0472-META2_0475*	Transposase of ISMex1, IS3 family (ORF 1)/conserved hypothetical protein
META2	1,153,967	+1620 bp	Gain ISMex4; intergenic	*META2_1243-META2_1245*	Transposase of ISMex10, ISL3 family/transposase of ISMex4, IS1380 family
**Unmapped Archival AM1 DNA**					
**Size (bp)**	**Location(s)**	**Composition**			
11908	?	Porin protein, transcriptional regulator (AraC) protein, conjugative relaxase domain protein, sodium/hydrogen exchanger, TraG homolog			
8419	META1_4345/META2_0137	TonB-dependent receptor/siderophore receptor protein, hypothetical proteins			
8000	META1_1083	Sodium/calcium exchanger, hypothetical proteins			
5207	META2_0137	Cold shock protein A (*cspA*), metallophosphoesterase, plasmid stabilization system, addiction module antidote protein, cobyrinic acid ac-diamide synthase, stability/partitioning determinant, hypothetical proteins			
2423	p2META_0017	Oxidoreductase molybdopterin binding protein, sulfite:cytochrome c oxidase subunit B, hypothetical proteins			
**35957**	**Total bp**				

Mutations were identified by comparing the Archival genome to a previously sequenced Modern reference [21]. By comparing the mutational state (Archival or Modern) at each site to other previously sequenced strains of *M. extorquens* (see Methods), all but two mutations can be unambiguously traced as having occurred on the branch from the Ancestral to Modern AM1 (Figure 1). The effect of nonsynonymous mutations on coding regions are highlighted in bold and italic.

Some Modern mutations are likely to have far-reaching physiological consequences. Certain mutations target highly pleiotropic genes such as *rplJ*, which encodes the ribosomal subunit protein L10; *rpoB*, the beta subunit of RNA polymerase; and *recG*, the primary DNA helicase involved in recombination repair and other functions. The insertion of mobile elements might also have altered physiology in the evolution of Modern AM1, jumping into several putative protein-coding genes. Still other mutations resulted in the loss of a substantial amount of DNA from Archival to Modern AM1. Deleted regions were typically flanked by insertion sequences and other low complexity DNA, but in most instances we could infer the likely genomic location and content of these deletions (Table 1). Those genes deleted in Modern AM1 are predicted to perform a variety of functions, and appear in some instances to be homologs of genes found only in distantly related members of the *Methylobacterium* genus. Further insights into the extensive loss of DNA in Modern AM1, as well as the functional and evolutionary consequences of Modern mutations, will require considerable future work. Still, these results clearly show that the Modern AM1 lineage has undergone a substantial degree of evolutionary divergence and domestication over fifty years of growth and storage in the lab.

### Archival is faster and fitter under most standard growth conditions

To begin to explore the phenotypic differences arising in Modern AM1, we compared the performance of Modern and Archival strains grown on standard growth substrates in “Hypho” minimal medium. Two primary metrics were used to assess growth: specific growth rate, determined from the increase in optical density (OD_600_) for a particular strain/condition using a custom-built, high-throughput microbial culturing system and analysis software [27,44]; and relative fitness, which analyzes performance over multiple phases of bacterial growth (*i.e*. lag, exponential, and stationary) using a head-to-head competition of strains in co-culture [30].

**Figure 2:**
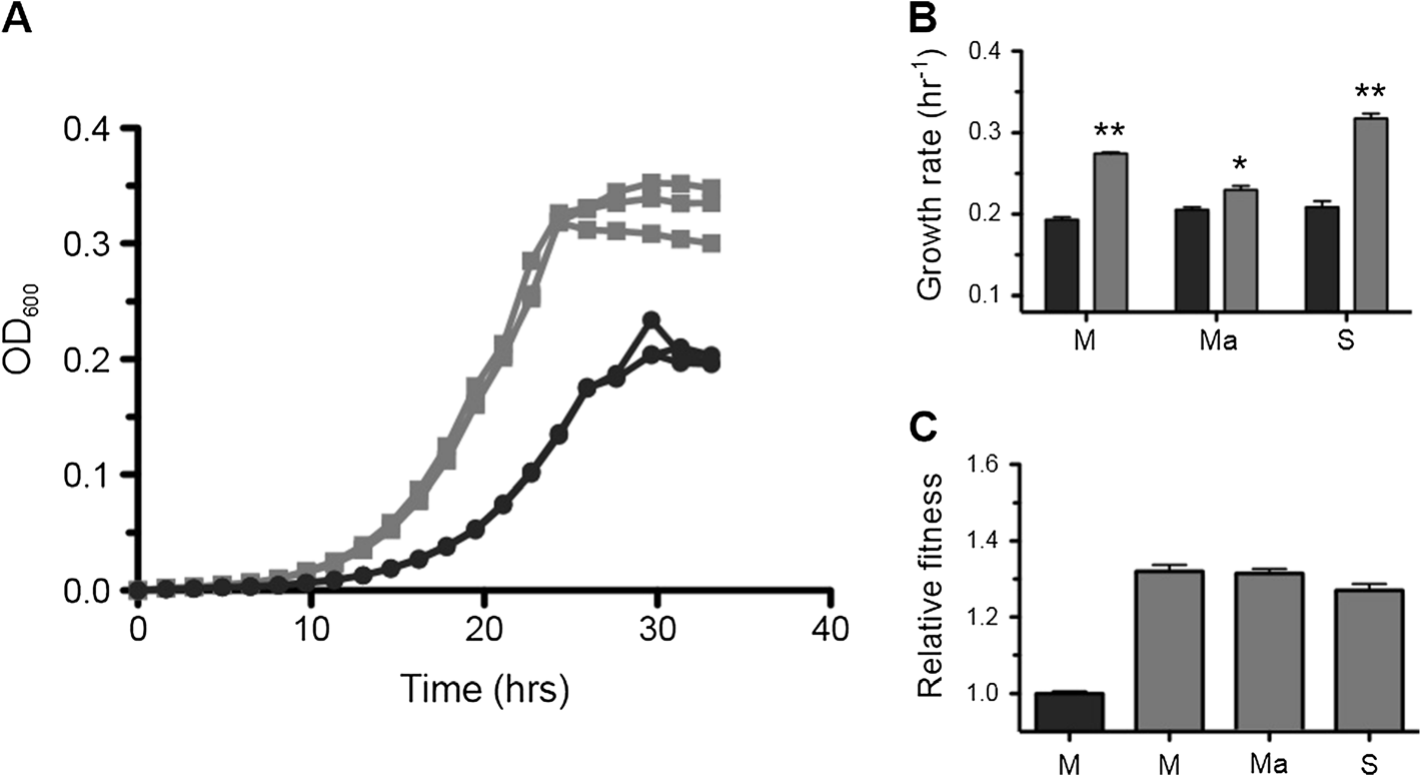
**Archival outperforms Modern AM1 under standard growth conditions. A)** Representative growth curves showing the increase in OD_600_ over time of Modern (black circles) and Archival AM1 (gray squares) cultured using 3.5 mM succinate in 48-well plates. **B)** Growth rates calculated from the exponential phase of cultures grown on methanol (M), methylamine (Ma), or succinate (S) as a carbon source. Significant growth differences between Modern and Archival were calculated using a two-tailed, unpaired *t* test, and are marked by single (p < 0.05) and double asterisks (p < 0.01). **C)** Fitness of Archival AM1 measured via a head-to-head competition mixed in co-culture with a fluorescently labeled Modern reference. A control growth consisted of unlabeled Modern (black) versus the fluorescent Modern reference grown on M. All other bars (gray) show Archival fitness relative to Modern grown M, Ma, and S. Values are the mean plus SEM of growth rates or fitness values calculated from three or more biological replicates (see Methods).

Our initial hypothesis was that Modern AM1 would outperform the Archival strain, owing to an increased likelihood of mutations in Modern AM1 that could facilitate adaptation to laboratory conditions. However, contrary to our expectations, we found that the Archival strain was both faster and fitter under most conditions tested. The Archival strain was considerably faster growing on the C_1_ compounds methanol (42%) and methylamine (12%), as well as the multi-C substrate succinate (52%; Figure 2A and B). In head-to-head fitness competitions, the Archival strain showed roughly a 30% advantage across the substrates tested (Figure 2C), suggesting that analyses of growth rate and fitness are not entirely correlated. Nonetheless, these results show that the Archival strain outperforms Modern under most standard growth conditions, and suggest that the Modern lineage became slower and less fit during its time in the lab.

### Modern outperforms the Archival AM1 on nutrient broth

We next sought to compare Modern and Archival AM1 using less traditional growth substrates. In contrast to “Hypho” minimal medium, nutrient broth (NB) is a rich medium composed of partially digested proteins (peptone), but its exact nutritional components are largely undefined. Today, NB is rarely used as a growth substrate for *Methylobacterium*, and is used almost exclusively to support growth in co-cultures with *E. coli* during conjugal matings for genetic manipulation. It is possible, however, that Modern AM1 was more frequently cultured on NB in the past and may have adapted to growth on this medium. To test this hypothesis, we assessed the growth and fitness of Modern and Archival AM1 cultured on NB.

**Figure 3:**
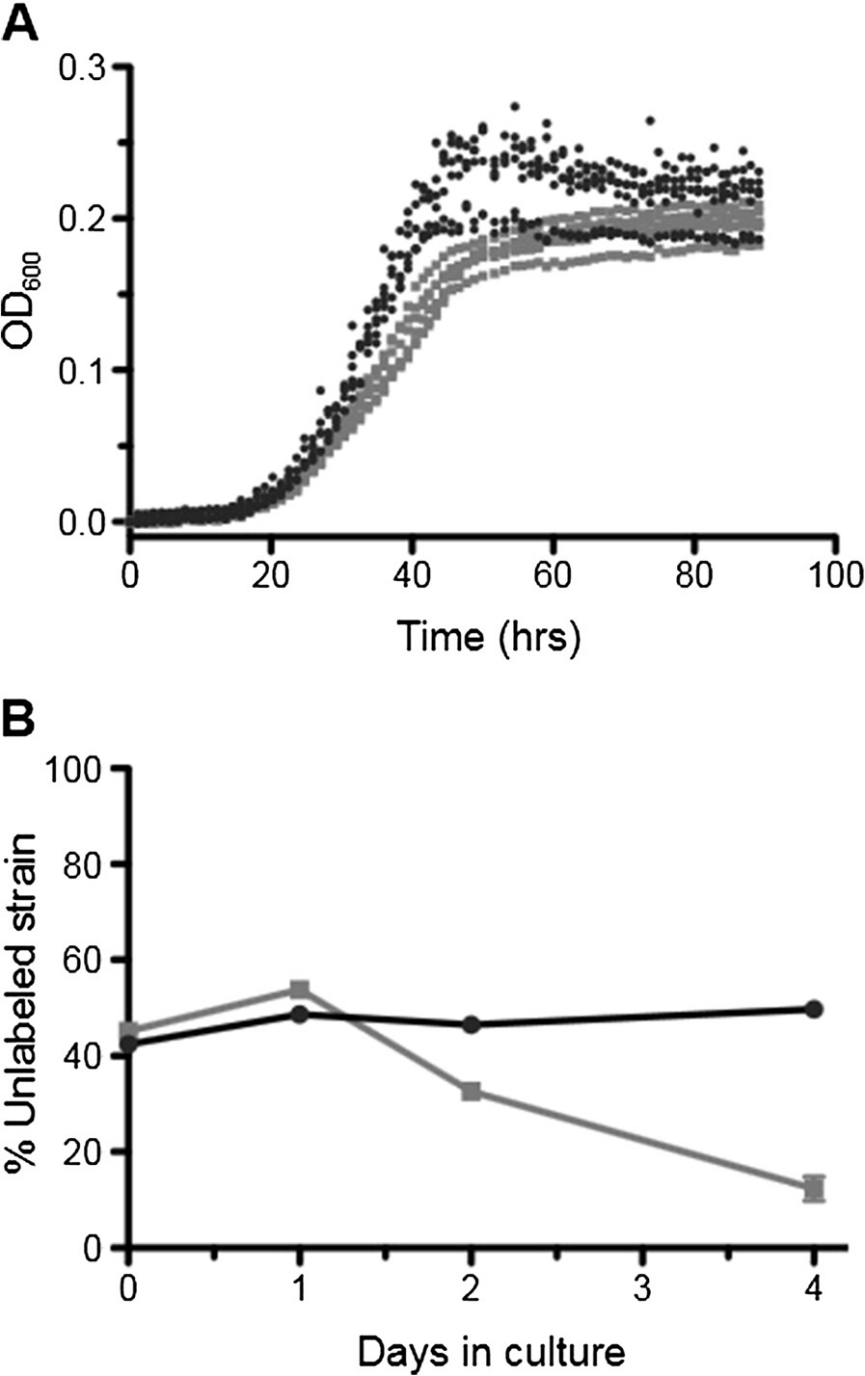
**Modern outperforms Archival AM1 when grown on nutrient broth. A)** Representative growth curves of Modern (black circles) and Archival (gray squares) AM1 grown on nutrient broth (NB). Note that growth - particularly for the Archival strain - slows considerably during late exponential phase, signifying density-dependent growth inhibition. **B)** Change in the proportion of either Modern or Archival AM1 mixed in co-culture with a fluorescently labeled Modern reference as measured by flow cytometry. Values represent the mean plus SEM of at least three biological replicates grown in 48-well plates **(A)** or flasks **(B)**.

We found over multiple different growth experiments that Modern AM1 consistently outperformed Archival on NB, although both strains grew more poorly than on Hypho medium. Modern displayed a clear growth advantage on NB in 48-well plates (Figure 3A), which could be traced in part to its relative insensitivity to perturbations arising during the later stages of NB growth. While monitoring the increase in OD_600_ over time, we observed that NB cultures tended to slow down during the later stages of exponential growth, and that the Archival strain was hindered to a greater extent than Modern. This suggested that Modern may have adapted to yet unknown components of growth in NB; however, due to this decrease during late exponential phase, we were unable to accurately assess differences in specific growth rate, and sought instead to quantify performance using a head-to-head competition of Modern and Archival co-cultures.

The performance of Archival AM1 co-cultured with a fluorescently labeled Modern reference was monitored over the course of several days during growth in NB flasks. Co-cultures were sampled periodically to monitor changes in the ratio of nonfluorescent (Archival) to fluorescent (Modern) cells using flow cytometry. These results suggest that the Archival AM1 holds an early growth advantage in NB that quickly decreases from 1 to 4 days until the strains reach stationary phase (Figure 3B). As a control, a co-culture of nonfluorescent Modern mixed with the same fluorescent Modern reference showed little change over the course of the experiment. Using the ratio of nonfluorescent to fluorescent cells at the start and the end of one growth cycle, we can calculate the fitness of the Archival AM1 relative to Modern assuming a 64-fold (2^6^) increase in the population (see Methods), and find that Archival is only 61% as fit as Modern during growth on NB. Overall, the improved performance of Modern on NB is suggestive of adaption, either specifically to this medium, or to yet unknown but similar growth conditions.

### Archival and Modern are similar in terms of long-term growth and storage

Another dimension in which AM1 may have adapted to life in the lab is through improved performance during long-term growth and storage. We compared the Modern and Archival strains grown for extended periods both in flasks and on plates by creating co-cultures of each strain with a fluorescently labeled Modern reference, and monitoring the change in fluorescent to non-fluorescent cells over time using flow cytometry. Growth in flasks was performed over 14 days with continual shaking using succinate as a growth substrate, while growth on methylamine plates was carried out at 30°C for 4 days, followed by up to 60 days at 4°C to simulate long-term storage.

**Figure 4:**
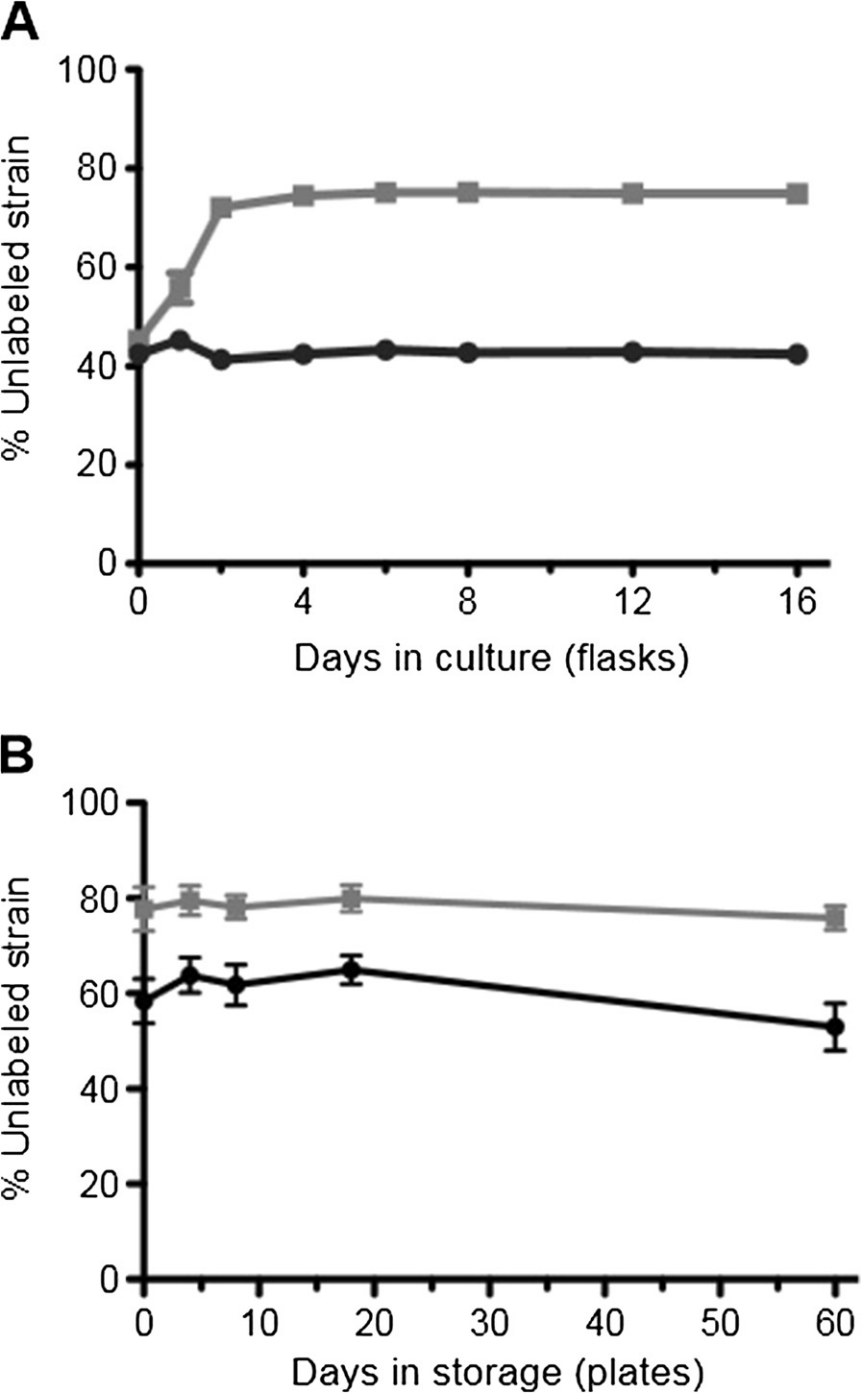
**Equivalence of AM1 strains during tests of long-term growth and survival.** Co-cultures were created by mixing either Modern (black circles) or Archival AM1 (gray squares) with a fluorescently labeled Modern reference, and the change in unlabeled versus fluorescent cells was monitored over time using flow cytometry. **A)** In continually shaken flasks with succinate, the Archival strain increased in frequency over the first two days of growth and maintained this advantage over Modern over time. **B)** Similarly, Archival increased in frequency during four days of growth on methylamine agar plates (not shown), and maintained this frequency during long-term storage at 4°C. Values represent the mean plus SEM of the percent unlabeled cells measured in three replicate co-cultures.

Here, Modern and Archival AM1 were roughly equivalent in terms of growth and survival during stationary phase. In flasks, the ratio of unlabeled Archival cells to labeled Modern cells was steady for 12 days after the initial 2 days of exponential growth (Figure 4A), while the unlabeled Modern control remained unchanged for the duration of the experiment. Archival held a similar advantage during storage at 4°C on agar plates for up to 60 days (Figure 4B). Collectively these results suggest that, at least under these conditions tested, the extent to which laboratory domestication improved the long-term growth and survival of AM1 is limited, and that the major difference between these strains lies in decreased exponential phase growth in Modern under standard conditions, and slightly increased growth on NB.

### Growth of AM1 is significantly hampered by selection for rifamycin resistance

To explain the decreased performance of Modern AM1 under most growth conditions, we returned to the genomic changes identified in this lineage. All but one of the 29 mutations arising in Modern AM1 can be attributed to unintended laboratory domestication; this single exception, however, was central to the development of Modern AM1. In 1984, Fulton and colleagues selected for rifamycin resistance (Rif^R^) to facilitate genetic manipulations in AM1 using conjugative, tri-parental matings [45]. Across numerous systems, mutations conferring Rif^R^ most often occur in the beta subunit of RNA polymerase – encoded by *rpoB* – and typically give rise to fitness tradeoffs between survival in the presence of antibiotics versus decreased growth in their absence [46]. Compared to the Archival strain, Modern AM1 has two mutations to the *rpoB* locus (Table 1), one of which (Q521R) falls in a region that confers Rif^R^ in a variety of other organisms [47], while the effect of the other (Q1081R) is yet unknown. At both positions the amino acid state of the Archival strain is universally shared with other non-AM1 strains of *M. extorquens*, suggesting that these mutations arose exclusively in the Modern lineage. Thus, these mutations to *rpoB*, particularly Q521R, are strong candidates for decreased overall performance in Modern AM1: offering resistance in the presence of Rif but slower growth in its absence.

To explore the potential costs associated with antibiotic resistance, we recapitulated the evolution of Rif^R^ in independent replicate cultures of the Archival strain. Out of 36 independent populations grown to saturation, only 7 produced a small number of spontaneous, resistant colonies when plated on Rif agar plates. Each independent population was streaked to purity, analyzed in terms of growth rate on succinate (with no antibiotic), and sequenced along with the Modern and Archival strains at the *rpoB* locus.

Independent experiments recapitulating Rif^R^ in the Archival strain all selected for mutations to *rpoB*, and all resulted in decreased growth in the absence of antibiotic. Upon sequencing *rpoB* from the 7 newly evolved Archival isolates, we found that Rif^R^ was always associated with mutations to *rpoB*. One of these strains (CM4022) acquired the exact same nonsynonymous change that occurred in Modern evolution, corroborating our hypothesis that the Q521R mutation (from Archival to Modern) is causal in conferring Rif^R^ to Modern AM1. Indeed, all mutations observed fall within a region of *rpoB* that is commonly mutated to confer Rif^R^ across a variety bacteria [47].

**Figure 5:**
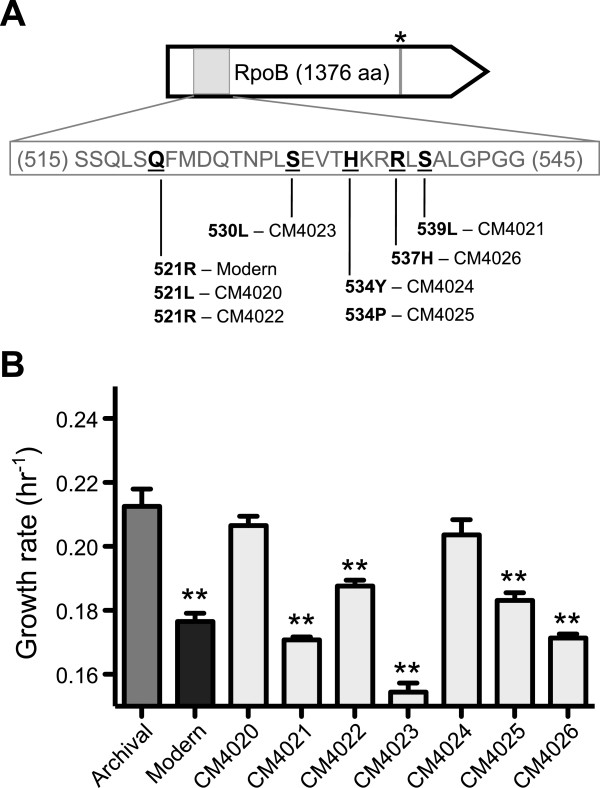
**Mutations associated with rifamycin resistance hinder AM1 growth. A)** Spectrum of mutations to the RNA polymerase beta subunit (RpoB) during past and current selection for rifamycin resistance (Rif^R^). Modern AM1 was selected for Rif^R^ in 1984 [45] and acquired two mutations to RpoB: Q521R, and Q1081R (denoted by an asterisk). By recapitulating selection for Rif^R^ in replicate Archival populations, we identified a number of other RpoB mutations putatively associated with Rif^R^. **B)** The effect of Rif^R^ mutations on growth rate in the absence of antibiotic. Values represent the mean plus SEM of four biological replicates grown in 48-well plates with succinate. Strains that were significantly slower than Archival are marked with asterisks (p < 0.01, one-way ANOVA with Tukey post-hoc test).

The spectrum of *rpoB* mutations across Rif^R^ Archival isolates displayed highly variable effects on growth rate in the absence of antibiotic. Compared to their Archival ancestor, several Rif^R^ isolates show very little decrease in performance when grown on succinate, while other isolates slow to near Modern levels, and still others grow substantially worse than Modern (Figure 5B). Interestingly, the CM4022 isolate that perfectly recapitulated the change from Archival to Modern AM1 (Q521R) was slightly faster than Modern, suggesting perhaps that other mutations further hamper growth in the Modern lineage. We note, however, that a direct comparison of this strain is difficult given that many other mutations and mutational interactions were likely present in Modern AM1 during the original selection for Rif^R^. Nevertheless, these results demonstrate that selection for Rif^R^ can substantially hinder growth of the Archival strain in the absence of antibiotic, and that this single researcher-imposed event – not laboratory domestication - is the major mechanism by which Modern AM1 became slower growing in the lab.

## Discussion

This work highlights the surprising extent to which *M. extorquens* AM1 has inadvertently diverged during fifty years of growth and storage in the lab. Compared to an Archival AM1 strain, our Modern AM1 stock was slower growing under most standard laboratory conditions, and one mechanism to account for this decrease is in past selection for rifamycin resistance. Indeed, the recapitulation of Rif^R^ in independently evolved populations of the Archival strain frequently, but not always, led to a tradeoff between survival in the presence of antibiotic and decreased growth in its absence. Upon sequencing the Archival genome, we identified some 29 mutations that have accrued in the Modern AM1 lineage, including a number of single nucleotide polymorphisms, small insertions and deletions, the proliferation of mobile elements, and the loss of some 36 kb of DNA. Though the full impact of these mutations for improving growth on NB or other conditions has yet to be revealed, it is clear that Modern AM1 has diverged substantially through laboratory domestication and changes stemming from selection for antibiotic resistance.

At first glance, it was not immediately clear why Modern AM1 had become slower and less fit under standard growth conditions. Our initial hypothesis was that Modern would outperform the Archival strain due to the acquisition of mutations that optimized growth or survival in the laboratory, but our results suggested the contrary. Alternative hypotheses to account for decreased performance in Modern might include the chance acquisition of one or more mutations that are deleterious for growth, or that selection for mutations that improve growth under certain laboratory conditions generated tradeoffs in others. In the latter case, a strong candidate emerged in tradeoffs generated by selection for antibiotic resistance. Antibiotic resistance in organisms often carries with it a fitness cost in the absence of antibiotic, and this has been documented in related strains to our Modern AM1 [48]. We discovered two mutations to *rpoB* in our Modern strain: one that is hypothesized to confer resistance (Q521R), while the effect of the other is yet unknown but might serve a compensatory function [49]. Reselecting for Rif^R^ in 7 independent populations of Archival AM1 resulted in a variety of mutations to *rpoB* with variable effects on growth in the absence of antibiotic. One strain in particular (CM4022) perfectly recapitulated the Q521R mutation in Modern AM1, and yet showed slightly faster growth than Modern despite lacking any other known changes. While a direct comparison of this strain and the original selection for Rif^R^ in Modern AM1 is complicated by the presence of other mutations in the genetic background of the latter, these results strongly suggest that selection for Rif^R^ was the major factor affecting growth in the Modern strain. Given the large number of genomic changes identified in the Modern lineage (n = 29), it is somewhat surprising that the only mutation attributed to purposeful laboratory selection, and not domestication, accounts for much of the phenotypic divergence observed between the Archival and Modern strains.

Though the exact circumstances under which Modern AM1 evolved cannot in most cases be ascertained, we can at least hypothesize as to the general factors that might have played a role. Prior to the widespread use of −80°C freezers, long-term storage of AM1 cultures was accomplished using Hypho agar slants with methanol or methylamine [18,50], most often under refrigeration. A study of *Salmonella*[4,5] and *E. coli*[6-8] archived for decades under similar conditions revealed a number of genomic and physiological changes that aid in survival during long-term stationary phase. These conditions often select for mutants with a “growth advantage in stationary phase” (GASP) phenotype associated with increased catabolism of amino acids and other small organic compounds, as well as the ability to outcompete “younger” cultures [51]. Although a GASP-like phenotype was not observed in the two test environments with which we compared Modern versus Archival AM1, further experiments might reveal conditions in which Modern AM1 has adapted to other facets of laboratory growth or storage. One particularly interesting direction is in the advantage of Modern on NB, which may have evolved specifically to this medium or merely correlate with improvements in other conditions, such as the ability to survive long-term growth with limiting nutrients. Along these lines, we analyzed the growth of Rif^R^ strains (CM4020-26) on NB and found that they performed equally as well, if not worse, than their Archival parent strain (data not shown). This suggests that improved growth of Modern on NB is due to one or more mutations that arose during laboratory domestication, not through the *rpoB* mutation associated with Rif^R^.

Between long periods of storage, Modern AM1 may have also adapted to yet unknown growth conditions. The specific components of growth media can act as a strong selective pressure in microbial cultures [52-54], and over many years Modern AM1 experienced both numerous variants of minimal media, as well as occasional growths in rich media like NB. Large populations of microorganisms competing for limiting resources can create strong selective pressures for increased growth rate, which may have accrued in Modern AM1 only to be partly nullified due to later tradeoffs with Rif^R^. It is also possible that some mutations were fixed not through selection for improved performance in laboratory conditions, but rather via genetic drift stemming from practices that result in extreme population bottlenecks, such as colony picking. Given the complex and overall vague history of Modern AM1 in the lab, reconstructing the exact mechanisms that drove its evolution might be best accomplished by studying other “offshoots” of the laboratory-maintained Modern lineage, and by characterizing the selective effect of Modern mutations in the Archival genetic background using allelic exchange.

## Conclusions

The laboratory environment affords researchers with a great degree of control over experimental variables: from reagents and protocols, to the genotype of their model microbe, and the environment in which it lives in. Examples of laboratory domestication, however, highlight the difficulty of maintaining high quality microbial stocks. Mutations can jeopardize the integrity of microbial stocks and, given time, lead to spurious and inconsistent results stemming from the evolutionary divergence of strains. Even purposeful laboratory experiments intended merely to select for antibiotic resistance or otherwise alter the genetic background of strains can have unanticipated pleiotropic effects. Thus, extra care should be taken to ensure that experimental findings from strains are reproducible and consistent over time. For those stocks in which divergence has already occurred, these microbes offer a unique opportunity to explore genetic and phenotypic changes resulting from complex evolutionary processes at work in the lab.

## Methods

### Bacterial strains & growth conditions

Strains relevant to this study were as follows. Our Modern AM1 strain was derived from a pink, “wildtype” *M. extorquens* AM1 (CM501) described elsewhere [23]. A sample of lyophilized Archival AM1 (renamed CM3944) was acquired from the National Center of Industrial Food and Marine Bacteria (NCIMB #9133, Aberdeen, Scotland), grown to saturation, and frozen at −80°C in 8% DMSO. To limit cell clumping and reduce noise in OD_600_ measurements during analyses of growth rate, we utilized a strain of Modern AM1, CM2720, that lacks genes for cellulose biosynthesis [27] without a loss of growth or fitness. For competitions in co-culture, a fluorescently labeled Modern reference (CM1175) was constructed by placing the red fluorescent protein *mCherry* under control of a constitutive *P*_*tac*_ promoter at the *katA* locus [30]. Isolates from each of seven Archival populations selected for rifamycin resistance (described below) were numbered CM4020 through CM4026.

Standard growth conditions utilized a modified version of Hypho minimal medium consisting of: 100 mL phosphate salts solution (25.3 g of K_2_HPO_4_ plus 22.5 g Na_2_HPO_4_ in 1 L deionized water), 100 mL sulfate salts solution (5 g of (NH_4_)_2_SO_4_ and 2 g of MgSO_4_ • 7 H_2_O in 1 L deionized water), 799 mL of deionized water, and 1 mL of trace metal solution [55]. All components were autoclaved separately before mixing under sterile conditions. Carbon sources added just prior to inoculation in liquid minimal media consisted of 20 mM methanol, 3.5 mM sodium succinate, or 20 mM methylamine hydrochloride. Growths in 48-well microtiter plates consisted of Hypho medium plus the appropriate carbon source to a volume of 640 μL. Agar plates consisted of growth medium plus either 125 mM succinate or 100 mM methylamine and were autoclaved with 1.6% w/v agar. Difco nutrient broth (Becton, Dickson, and Company, Franklin Lakes, NJ) was prepared according to the manufacturer’s guidelines.

All growth regimes consisted of three phases consisting of inoculation, acclimation, and experimentation growths. All strains were stored in vials at −80°C in 8% DMSO; growths were initiated by transferring 10 μL freezer stock into 10 mL of Hypho medium with methanol. Upon reaching stationary phase (~2 days at 30°C with shaking), cultures were transferred 1:64 into the appropriate medium and vessel to be tested, allowed to reach saturation in this acclimation phase, and diluted 1:64 again into fresh medium for the measured (experimental) growth.

### Measurements of specific growth rate and relative fitness

The increase in OD_600_ for strains grown in 48-well microtiter plates was measured using an automated, robotic culturing and monitoring system [27,44]. The specific growth rate of cultures was calculated from the log-linear growth phase using custom-designed growth analysis software [27]. Growth rates reported for each strain and condition are the mean plus SEM calculated from triplicate biological replicates, unless otherwise noted. Exogenous cellulase enzyme from *Aspergillus niger* (Sigma-Aldrich, St. Louis, MO) was added to the medium to a final concentration of 0.1 mg/mL to further minimize cell clumping and facilitate more accurate measurements of OD_600_ over time (SMC, unpublished).

Fitness measurements – which encapsulate the lag, exponential, and stationary phases of growth – were performed using a head-to-head competition of strains grown in co-culture [30]. Modern and Archival AM1 were competed against a fluorescently labeled Modern strain (CM1175) expressing mCherry [30]. Co-cultures were prepared by mixing test strains with the fluorescent Modern reference in roughly equal optical densities. A sample of this co-culture prior to competition was diluted in 8% DMSO and stored at −80°C; the rest was diluted 1:64 into 640 μL Hypho medium plus carbon in 48-well microtiter plates and incubated with shaking at 30°C for 1 growth cycle. A sample of co-culture after competition was frozen for later analysis using flow cytometry.

The ratio of labeled to unlabeled cells in co-cultures before (R0) and after (R1) competition was measured using a BD LSR Fortessa flow cytometer with an HTS attachment (BD Biosciences, San Jose, CA). Both forward and side scatter settings were set to 300 V to account for the small size of bacterial cells [56], and the flow-rate was adjusted to the lowest setting to more accurately identify events (cells) in dilute co-cultures. The fitness (W) of strains relative to the labeled Modern reference was calculated using the following formula, which assumes a 64-fold expansion of cells following 6 doublings per growth cycle:

$$W=\log\;\left(\frac{R_{1^{\cdot }}64}{R_{0}}\right)/\log\left(\frac{\left(1-R_{1}\right)\cdot 64}{1-R_{0}}\right)$$

### Analysis of long-term growth and storage

To assess the ability of strains to withstand extended periods of stationary phase growth in flasks, Modern and Archival AM1 were mixed in co-culture with the fluorescent Modern reference, and the ratio of labeled to unlabeled cells was measured periodically using flow cytometry. Flasks possessing succinate were sealed to limit evaporation, and at the conclusion of the experiment co-cultures were streaked onto nutrient agar plates to check for contamination. Growth and survival on plates was measured using a similar experimental design: co-cultures were spread onto Hypho plus methylamine plates, grown for 4 days at 30°C, and then stored at 4°C for up to 60 days with periodic sampling.

### Recapitulation of Rif resistance in AM1

The re-evolution of Rif resistance in Archival AM1 was performed using 36 replicate lineages grown from single colonies in 48-well plates. After 2 days growth in liquid Hypho medium plus succinate, cultures were plated without dilution onto Hypho agar plus succinate plates containing 50 μg/mL Rif. After 5 days of growth, Rif^R^ colonies were obtained from 7/36 cultures and streaked twice more to ensure the purity of clonal isolates (CM4020-4026). PCR amplification plus sequencing was used to assess mutations to the *rpoB* locus and the growth rate of strains on succinate was determined as described above.

### Whole-genome sequencing of Archival AM1

Preparation of Archival genomic DNA was performed as described in [57]. Briefly, an Archival cell pellet was lysed using bead beating (MP Biomedicals, Solon, OH) and digested using heat, SDS and Triton-X100 detergents, proteinase K, and RNase A. Cell debris was precipitated with CTAB/NaCl, removed using a phenol/chloroform/isoamyl alcohol extraction, and genomic DNA recovered using an isopropanol precipitation. Illumina HiSeq2000 sequencing was performed out-of-house using GENEWIZ, Inc (South Plainfield, NJ). A total of 23,660,656 reads were generated, and 98.2% of these were mapped against the Modern AM1 reference [21] using breseq v0.21 [58] with Bowtie 2 version 2.0.0-beta7 [59]. To identify DNA lost in Modern AM1 evolution, reads that were unmapped using breseq were analyzed using ABySS v1.3.4-kmer-96 [60] for *de novo* assembly of contigs,. A full *de novo* assembly of the Archival genome was also performed in ABySS for comparison against breseq. Contigs from ABySS were further assembled and curated using contig assembly and BLAST in Geneious [61]. To determine whether mutational differences occurred in either the Modern or Archival lineage, we compared the mutational state (Archival or Modern) at each site to other previously sequenced strains of *M. extorquens* using both BLAST and analyses of conserved gene synteny using the Methyloscope project in MicroScope [62]. Mutations were identified as “derived” in the Modern lineage if the Archival state was consistently shared across related strains, to the exclusion of Modern AM1. Related strains included *M. extorquens* PA1 (GenBank Assembly ID: GCA_000018845.1), CM4 (GCA_000021845.1), DM4 (GCA_000083545.1), and DSM 13060 (GCA_000243435.2). For all loci but two, the Archival state was universally conserved; the only exceptions were for genes with little to no homology outside of Modern and Archival AM1.

## Availability of supporting data

Whole-genome sequencing data of the Archival AM1 strain has been deposited to the NCBI Sequencing Read Archive (SRR1046370).

## Abbreviations

AM1: *Methylobacterium extorquens* AM1; C1: One-carbon; multi-C: multi-carbon; OD600: Optical density; NB: Nutrient broth; RifR: Rifamycin resistance.

## Competing interests

The authors declare that no competing interests exist.

## Authors’ contributions

Designed experiments: SMC, KSX, and CJM. Performed experiments: SMC and KSX. Wrote and edited the manuscript: SMC, KSX, and CJM. All authors read and approved of the final manuscript.
